# Human hair follicle equivalents *in vitro* for transplantation and chip-based substance testing

**DOI:** 10.1186/1753-6561-5-S8-O7

**Published:** 2011-11-22

**Authors:** R Horland, G Lindner, I Wagner, B Atac, S Hoffmann, M Gruchow, F Sonntag, U Klotzbach, R Lauster, U Marx

**Affiliations:** 1 TU Berlin, Institute for Biotechnology, Faculty of Process Science and Engineering, 13355 Berlin, Germany; 2 Fraunhofer IWS Dresden, 01277 Dresden, Germany; 3 TissUse GmbH, 15528 Spreenhagen, Germany

## Introduction

The ability to create an organoid, the smallest functional unit of an organ, *in vitro* across many human tissues and organs is the key to both efficient transplant generation and predictive preclinical testing regimes. The hair follicle is an organoid that has been much studied based on its ability to grow quickly and to regenerate after trauma. Replacing hair lost due to pattern baldness or more severe alopecia, including that induced by chemotherapy, remains a significant unmet medical need. By carefully analyzing and recapitulating the growth and differentiation mechanisms of hair follicle formation, we recreated human hair follicles in tissue culture that were capable of producing a hair shaft and revealed a striking similarity to their *in vivo* counterparts. Extensive molecular and electron microscopy analysis were used to track the assembly of follicular keratinocytes, melanocytes and fibroblasts into the final hair shaft producing microfollicle architecture. The hair follicle generation process was optimized in terms of efficiency, reproducibility and compliance with regulatory requirements for later transplantation. In addition, we developed a procedure to integrate the *de novo* created human microfollicles into our existing chip-based human skin equivalents for substance testing. This would allow the evaluation of the role of hair follicles in dermal substance transport mechanisms for cosmetic products. Finally, we describe the challenges and opportunities we are facing for first-in-man transplantation trials.

## Material and methods

Mesenchymal, ectodermal and neuro-ectodermal originated cells from dissected human hair follicles were isolated and expanded into multiple passages. Dermal papilla fibroblasts have been kept under low adherent culture conditions resulting in the formation of dermal papilla-like aggregates. These spheroids underwent extra cellular matrix protein coating which mimics basal membrane compositions and thereby retained their inductive properties. In subsequent co-culture procedures keratinocyte and melanocyte attachment to the spheroids was forced allowing further follicular development. Ultra-structural examinations by scanning electron microscopy and immunofluorescence staining of cryo-sectioned microfollicles were performed to reveal spatiotemporal development and to characterize hair follicle like structures. Microfollicles were further integrated into full skin equivalents by dermal surface application or by integration through micromanipulation.

## Results

**Figure 1:**
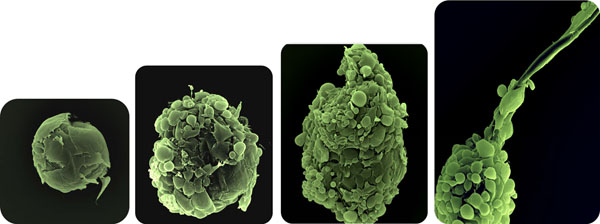
**Microfollicle formation**. SEM images taken from developing microfollicles. After adding keratinocytes and melanocytes to the culture medium a loosen attachment to the neopapilla is seen (A). Polarization of the early aggregate (B). Assembly, orientation and sheath formation (C); microfollicle with fiber production (D).

The formation of functional neopapillae (dermal papilla condensates) needs more than 48 hours. At day 7 of the condensation process the aggregation of cells is much more dense and the formation of an extracellular matrix becomes visible. After the addition of keratinocytes and melanocytes, the self-organizing microorganoids follow a stringent pattern of follicular-like formation by generating polarized segments, sheath formations and the production of a hair shaft – like fiber (Figure 1). Specific extracellular matrix proteins (e.g. Collagen IV, Chondroitin-4-sulfate, Versican) and defined mesenchymal and epithelial markers (e.g. Vimentin, CK15) were expressed. Selected proliferating (Ki67-positive) and apoptotic (TUNEL-positive) keratinocytes were detected in the outer microorganoid layers but also in the innermost dermal papilla-like aggregate. Microfollicles in skin equivalents self-organize in specific distance. Stainings show the integration of microfollicles below the epidermis.

## Conclusion

We show that the *de novo* formation of human microfollicles *in vitro* is accompanied by basic hair follicle like characteristics. The microfollicles can be used to study mesenchymal-epithelial-neuroectodermal interactions and for the *in vitro* testing of hair growth-modulating and pigmentary effects of substances. Hair follicles represent a long-term reservoir of topically applied substances. As the hair follicle is highly vascularized, it supports penetration of substances into the skin and further into the bloodstream. Since 2009 all cutaneous resorption experiments of the cosmetic industry in the EU have to be performed *in vitro. In vitro* testing of topically applied substances might therefore be performed with significantly enhanced validity by the incorporation of a microfollicle into a dynamic chip-based bioreactor containing a skin equivalent which mimics a physiological penetration route. With further improvements, the generated microfollicles embedded in an artificial skin model might also in future be used as improved implants for treating wound conditions. Eventually GMP-compliant manufactured human neopapillae might be used as implants for treating reduced hair conditions in humans.

